# Interactions between functional networks in Parkinson's disease mild cognitive impairment

**DOI:** 10.1038/s41598-023-46991-3

**Published:** 2023-11-17

**Authors:** Manuel Delgado-Alvarado, Vicente J. Ferrer-Gallardo, Pedro M. Paz-Alonso, César Caballero-Gaudes, María C. Rodríguez-Oroz

**Affiliations:** 1 Neurology Service, Hospital Sierrallana, 39300 Torrelavega, Spain; 2 Neurodegenerative Disorders Research Group, University Hospital Marqués de Valdecilla-IDIVAL, 39008 Cantabria, Spain; 3 Centro de Investigación Biomédica en Red de Enfermedades Neurodegenerativas (CINERNED), Madrid, Spain; 4 Basque Center on Cognition Brain and Language (BCBL), 20009 San Sebastian, Spain; 5 Ikerbasque, Basque Foundation for Science, 48009 Bilbao, Spain; 6 Neurology Department, Clínica Universidad de Navarra, Av. de Pío XII, 36, 31008 Pamplona, Navarra Spain; 7 Navarra Institute for Health Research (IdiSNA), 31008 Pamplona, Spain

**Keywords:** Parkinson's disease, Dementia

## Abstract

The study of mild cognitive impairment (MCI) is critical to understand the underlying processes of cognitive decline in Parkinson’s disease (PD). Functional connectivity (FC) disruptions in PD-MCI patients have been observed in several networks. However, the functional and cognitive changes associated with the disruptions observed in these networks are still unclear. Using a data-driven methodology based on independent component analysis, we examined differences in FC RSNs among PD-MCI, PD cognitively normal patients (PD-CN) and healthy controls (HC) and studied their associations with cognitive and motor variables. A significant difference was found between PD-MCI vs PD-CN and HC in a FC-trait comprising sensorimotor (SMN), dorsal attention (DAN), ventral attention (VAN) and frontoparietal (FPN) networks. This FC-trait was associated with working memory, memory and the UPDRS motor scale. SMN involvement in verbal memory recall may be related with the FC-trait correlation with memory deficits. Meanwhile, working memory impairment may be reflected in the DAN, VAN and FPN interconnectivity disruptions with the SMN. Furthermore, interactions between the SMN and the DAN, VAN and FPN network reflect the intertwined decline of motor and cognitive abilities in PD-MCI. Our findings suggest that the memory impairments observed in PD-MCI are associated with reduced FC within the SMN and between SMN and attention networks.

## Introduction

Parkinson’s disease (PD) is currently viewed as a complex neurodegenerative disease that involves several cerebral areas and neurotransmitter systems. Although motor symptoms are the hallmark of clinical diagnosis, non-motor symptoms play a prominent role in the course of the disease. For many patients, the most disruptive non-motor manifestations of PD are cognitive impairment and dementia, which reach a long-term prevalence of up to 80%^1^. Mild cognitive impairment (MCI) is highly prevalent in PD (PD-MCI) (mean 26.7%; range 18.9–38.2%)^2^ and is known to be a risk factor for PD with dementia^2,3^. Yet, PD-MCI is a rather heterogeneous condition, encompassing several subtypes of cognitive decline, thought to confer different risks of progression to dementia^4^. This heterogeneity reflects the diverse neuropathological processes of PD-MCI^5^ which have been most consistently associated with the progressive accumulation of α-synuclein, amyloid-β and pathologic tau species, particularly in frontal, temporal and cingulate areas^6^.

PD research has led to the characterization of two cognitive deterioration profiles: a fronto-executive dysfunction mainly driven by dopaminergic loss and manifesting as deficits in flexibility, planning, working memory and reinforcement learning; and a cholinergic cortical dysfunction leading to memory deficits and dementia (i.e., amnestic)^7^. However, these two profiles do not always capture the heterogeneous cognitive deterioration of PD-MCI. Instead, a range of cognitive impairments arises from the progressive and heterogeneous involvement of distinct neural networks, modulated by dopaminergic and cholinergic neurotransmitter systems^8^.

A wide body of evidence supports the existence of a hierarchically organized resting state network (RSN) system in which association networks serve higher-level cognitive functions^9^. Here, we use the seven major RSNs identified in Yeo et al.^10^ namely the visual (VN), sensorimotor (SMN), dorsal attention (DAN), ventral attention (VAN), limbic (LN), fronto-parietal (FPN) and default mode (DMN) networks, plus the striatum. As described below, four of these RSNs (SMN, DAN, VAN and FPN) have been implicated in the pathology seen in PD-MCI patients.

The FPN is a critical component in executive function and working memory^11^, and its main hubs suffer from dopamine denervation in PD^12^. Meanwhile, attentional orienting is driven by both the ventral attention network (VAN)^13^ and the dorsal attention network (DAN)^14^, which have also been associated with PD-MCI^15–17^. The orienting attentional system is associated with the cholinergic neurons of the basal forebrain, and the cholinergic neurotransmitter loss present in PD-MCI subjects has been linked to attention deficits in this system^11,18^. Finally, the SMN mainly serves primary motor functions, although it also coordinates with other cognitive networks. Although the role of the SMN in PD-MCI is an ongoing discussion. On one side, SMN disruption could be a reflection of motor impairments in PD-MCI patients, which is reflected in their scores in the "movement disorders society unified Parkinson’s disease scale” (MDS-UPDRS)^2,19^. On the other side, SMN disruptions have been related to impaired sensory integration for motor function in PD^20^. The SMN has also been associated with the fronto-executive dysfunction cognitive profile observed in PD, mainly impacting the FPN. For example, the SMN plays a crucial role in verbal short-term memory, coordinating with the fronto-temporal areas^21^. More evidence of the SMN cognitive role arises from disruptions in this network in association with cognitive impairment in PD and even in Alzheimer´s disease (AD)^22–24^.

Here, we focus on alterations in the RS functional connectivity (FC) between these RSNs in PD-MCI. Resting state functional connectivity (RSFC) can be defined as a significant temporal correlation between functionally related brain regions in the absence of any stimulus or task^25^. Methodological approaches to the analysis of RSFC have employed RS functional magnetic resonance imaging (fMRI). Studies of RSFC of the diseased brain have adopted a variety of methods, however, most attempt to determine whether there is an increase (i.e., higher synchronicity) or a decrease (i.e., lower synchronicity) within or between RSNs. Such studies are improving our understanding of the functional brain changes underlying PD-MCI. Conventional methods for analyzing data in RSFC fMRI studies cannot effectively capture and differentiate the shared underlying factors or conflicting processes that arise from distinct functional patterns observed in the brains of healthy and diseased individuals, using a data-driven approach. In the present work, we sought to use a recently implemented methodology that is ideal for examining the interaction between multiple brain systems or networks at the FC level. These FC patterns (i.e., FC-traits) represent different functional mechanisms between networks. Afterwards, we associate those between-network FC-trait features with the cognitive performance tests used for proper diagnosis and cognitive subtype classification in PD-MCI in line with Movements Disorders Society (MDS) criteria level II^26^. Although few studies have used MDS criteria level II^27^, it is thought that these criteria give better diagnostic sensitivity and specificity than criteria level I^26^. This is why in the present work we use criteria level I to infer which MCI cognitive domain is associated with the FC-traits. The data-driven methodology that we used here is termed *ConnICA,* which implements Independent Component Analysis (ICA) to extract robust independent FC-traits from a set of individual FC matrices^28,29^. An FC-trait represents a FC pattern between resting state networks in our FC matrices with a possible role in cognitive, motor, or clinical impairments.

We hypothesize that cognitive function deficits in PD-MCI would be driven by altered patterns of inter-connectivity between the brain networks. Specifically, the FC in networks such as the SMN, FPN, DAN and VAN would reflect such cognitive decline and would, therefore, capture the FC differences between PD-MCI and PD-CN. Here, we aim to extract independent FC-traits by applying *connICA* in PD-MCI patients, cognitively normal PD patients (PD-CN) and healthy controls (HC), to compare and disentangle key FC-traits that are altered in PD-MCI. Furthermore, we expect these specific connectivity patterns to be associated with performance on attention, executive function, memory, language, and visuospatial tasks.

## Results

42 PD patients (mean age 70.27 ± 6.32 years, 15 females) (23 PD-MCI and 19 PD-CN) and 21 HC (mean age, 67.52 ± 6.97 years, 9 females) remained from the 71 initial subjects after data quality control for motion or artifacts in the resting-state fMRI images. Table 1 depicts the sociodemographic and clinical characteristics of the groups. PD patients have higher scores than HC on the HADS scale, but this difference is not clinically relevant. PD-MCI patients were older than PD-CN patients (*P* = 0.017) and HC (*P* = 0.018), had fewer years of education than PD-CN (*P* = 0.003) and HC (*P* = 0.001) and higher scores in the MDS-UPDRS-III than PD-CN patients (*P* = 0.028). Cognitive assessments showed PD-MCI patients presented a multidomain deficit with higher deficits in attention and working memory, executive function, and memory domains than in language and visuospatial abilities (see Table 2 for cohort cognitive differences and Table S.1 for PD-MCI specific domain deficits).

**Table 1:** Sociodemographic and clinical characteristics of the cohort.

	PD-CN (n = 19)	PD-MCI (n = 23)	HC (n = 21)	P-values	PD-CN vs. PD-MCI	PD-CN vs. HC	PM-MCI vs. HC
Age (years)	66.58 ± 7	72.22 ± 5.43	68.65 ± 3.9	1.5 × 10^−2^ * (ANOVA)	5.4 × 10^−3^*	3.3 × 10^−1^	5.0 × 10^−3^*
Gender (male)	16 (80%)	12 (52.2%)	11 (52.4%)	1.1 × 10^−1^ (χ2)			
Education (years)	13.84 (ir 9.5–20)	8.96 (ir 6.5–10.5)	12.85 (ir 9–16)	1.8 × 10^−3^ (Kruskal Wallis)	3.0 × 10^−3^*	5.2 × 10^−1^	5.0 × 10^−3^*
Disease duration (years)	6.789 (ir 4–10)	9 (ir 4–13)	–	3.4 × 10^−1^ (Mann Whitney U)			
LEDD	947.77 ± 433.33	1089.86 ± 507.31	–	3.5 × 10^−1^ (T)			
MDS-UPDRS III	18.63 ± 8.51	26.36 ± 12.07	–	2.8 × 10^−2^* (T)			
MDS-UPDRS-III Axial score	1.684 ± 1.765	2.565 ± 1.805		1.1 × 10^−1^ (T)			
PDQ-39	20.74 (ir 10.5–26.5	33.391 (ir 20.5–45.00)	–	2.2 × 10^−2^* (Mann Whitney U)			
HADS depression	2.316 (ir 1–3.5)	4.217 (ir 2 – 6)	1.7 (ir 1 – 2.25)	3.0 × 10^−3^* (ANOVA)	3.3 × 10^−1^	2.0 × 10^−3^ *	3.4 × 10^−2^*
HADS anxiety	3.053 (ir 1.5–4)	4.869 (ir 2 – 7.5	3.8 (ir 1–5)	1.2 × 10^−1^ (ANOVA)			
HADS total	5.368 (ir 2–8.5)	9.087 (ir 5 – 12)	5.5 (ir 2 – 7)	1.6 × 10^−2^* (ANOVA)	9.1 × 10^−1^	2.3 × 10^−2^*	2.4 × 10^−2^*
H&Y stage	2.0526 (ir 2 –2.75	2.413 (ir 2– 3)	–	6.3 × 10^−2^ (Mann Whitney U)			

*HADS* Hospital anxiety and depression scale, *MDS-UPDRS* Movement disorders-Unified Parkinson’s Disease Rating Scale, *LEDD* Levodopa Equivalent Daily Doses, *ir* Interquartile range.

Asterisks indicate significant differences between groups.

**Table 2:** Cognitive characteristics of the cohort.

	PD-CN (n = 19)	PD-MCI (n = 23)	HC (n = 21)	ANOVAP-values	PD-CN vs. PD-MCI	PD-CN vs. HC	PD-MCI vs.HC
MOCA	25.947 ± 3.407	20.348 ± 3.4	26.4 ± 2.891	2.0 × 10^−7^*	2.0 × 10^−5^*	6.6 × 10^−1^	1.5 × 10^−6^*
MMSE	29.053 ± 1.129	27.13 ± 1.792	29.3 ± 0.865	1.7 × 10^−6^*	2.3 × 10^−4^*	4.5 × 10^−1^	1.2 × 10^−5^*
Attention and working memory	− 0.405 ± 0.560	− 1.536 ± 0.334	0.050 ± 0.905	5.0 × 10^−11^*	5.0 × 10^−10^*	6.8 × 10^−2^	1.0 × 10^−8^*
Span inverse	− 0.343 ± 0.598	− 1.099 ± 0.645	0.037 ± 1.040	4.0 × 10^−5^*	3.0*10^−3^*	1.7 × 10^−1^	8.3 × 10^−5^*
Digit and symbol	− 0.346 ± 0.704	− 1.973 ± 0.552	− 0.063 ± 1.073	1.2 × 10^−11^*	2.3 × 10^−10^*	1.7 × 10^−1^	7.0 × 10^−10^*
Executive function	− 0.310 ± 0.731	− 2.755 ± 1.363	0.037 ± 0.920	1.0 × 10^−12^*	1.0 × 10^−8^*	2.0 × 10^−1^	1.0 × 10^−9^*
Trail making test	− 0.284 ± 1.207	− 4.660 ± 2.533	0.030 ± 0.980	1.0 × 10^−12^*	5.0 × 10^−8^*	3.8 × 10^−1^	1.0 × 10^−9^*
Phonemic fluency	− 0.317 ± 0.687	− 1.053 ± 0.682	0.045 ± 1.074	2.0 × 10^−4^*	1.0 × 10^−3^*	2.2 × 10^−1^	2.0 × 10^−4^*
Memory	− 0.329 ± 0.914	− 1.35 ± 0.752	− 0.179 ± 0.712	2.0 × 10^−6^*	3.0 × 10^−4^*	2.9 × 10^−1^	8.0 × 10^−7^*
RAVLT	− 0.766 ± 1.288	− 1.708 ± 1.009	− 0.018 ± 1.038	2.0 × 10^−5^*	1.1 × 10^−2^*	5.0 × 10^−2^	3.0 × 10^−6^*
ROCF	0.107 ± 0.782	− 0.992 ± 0.924	− 0.075 ± 0.857	1.0 × 10^−4^*	1.0 × 10^−4^*	4.9 × 10^−2^	2 × 10^−3^*
Language	0.089 ± 0.838	− 1.175 ± 1.140	− 0.175 ± 0.872	1.0 × 10^−4^*	3.0 × 10^−4^	3.41 × 10^−1^	3.0 × 10^−3^*
Semantic fluency	0.053 ± 0.950	− 0.748 ± 1.035	− 0.179 ± 1.010	3.3 × 10^−2^			
Boston naming	0.125 ± 1.116	− 1.603 ± 2.024	− 0.171 ± 0.957	6.0 × 10^−4^*	2.0 × 10^−3^*	3.8 × 10^−1^	6.0 × 10^−3^*
Visuospatial abilities	− 0.656 ± 1.319	− 1.589 ± 1.875	0.032 ± 0.845	2.0 × 10^−3^*	7.5 × 10^−2^	5.8 × 10^−2^	9.0 × 10^−4^*
VOSP objects	− 0.377 ± 0.894	− 1.433 ± 1.297	0.065 ± 0.835	5.0 × 10^−5^*	4.0 × 10^−3^*	1.2 × 10^−1^	7.0 × 10^−5^*
VOSP numeric	− 0.936 ± 2.305	− 1.745 ± 3.033	− 0.002 ± 1.119	5.8 × 10^−2^			

Tests z-score group means and standard deviations. Z-scores from tests within the same cognitive domain are combined in one z-score that represents the whole domain (span inverse + digit and symbols = Attention and working memory). ANOVAs were computed for group mean comparisons, and t-tests were used for between-group comparisons. Asterisks indicate significant differences between groups.

*RAVLT* Rey Auditory Verbal Learning Test, *ROCF* Rey–Osterrieth Complex Figure, *VOSP* Visual Object and Space Perception Battery, *MMSE* Mini Mental State Examination, *MOCA* Montreal Cognitive Assessment.

**Figure 1:**
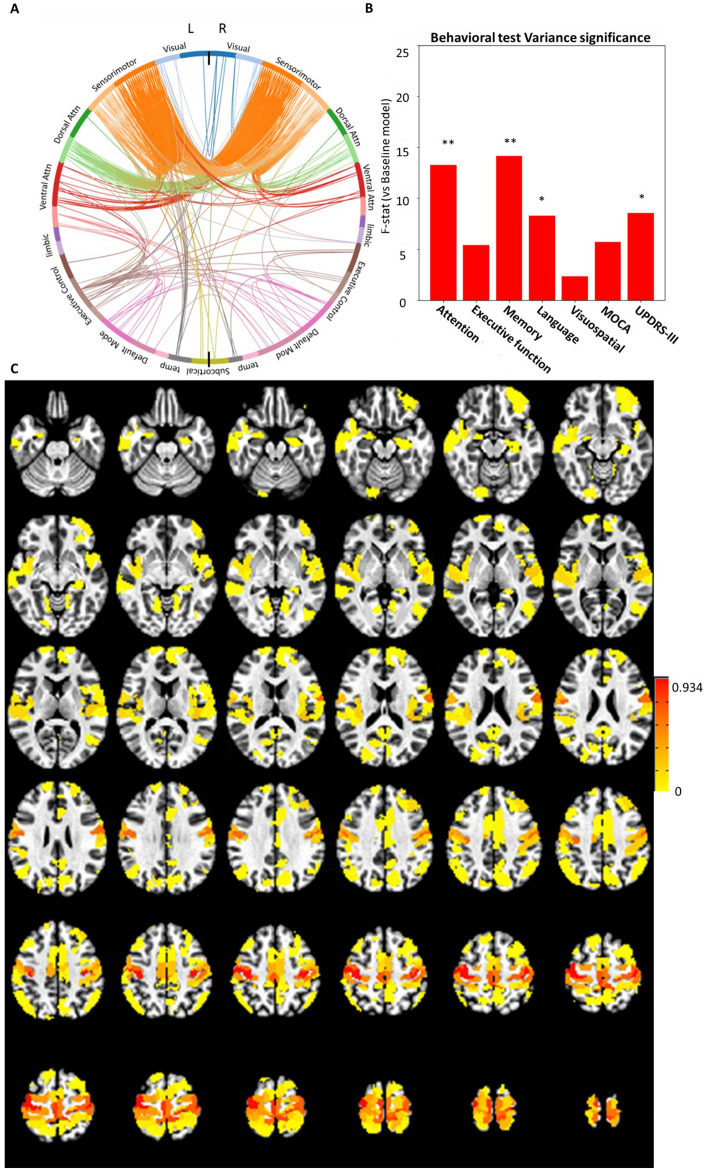
Characterization of the FC-Trait that showed significant differences between the groups. (**A**) Circular plot showing the 1% strongest connections in the left and right hemispheres, ordered by functional network. (**B**) The F-values obtained by comparing a baseline ANOVA model of the non-interest variables (i.e., age, MR sequence, average ENORM, total grey matter (TGM), total intracranial volume (eTIV)) with models adding one cognitive test. (**C**) Brain map showing the nodal strength of the 1% strongest connections calculated using the normalized FC values for the FC-trait.

Probabilistic-ICA was carried out extracting 65 independent FC-traits and their corresponding subject-specific connICA weights. Among these, only one FC-trait showed a statistically significant group effect (Table 3). Post-hoc pairwise comparisons between groups showed that this FC-trait (Fig. 1) differentiated PD-MCI from PD-CN and HC.

**Table 3:** Summary of the significant FC-trait.

FC-trait	SMN + +	DAN + +	LIMBIC-	VAN + +	DMN +	FPN +	VISUAL-	Striatum-
Main hubs	SMN: Bilateral precentral gyrus, Bilateral postcentral gyrus, Bilateral Somatomotor							
Statistics	GROUP rmANOVA *F*(8,64) = 8.63, *p* = 5.41 × 10^–4^* PD-MCI vs. PD-CN, *F*(4,41) = 23.09, *p* = 6.0 × 10^–6^* PD-MCI vs. HC, *F*(4,42) = 29.14, *p* = 6.0 × 10^–7^* PD-CN vs. HC, *F*(4,40) = 1.03, *p* = 3.1 × 10^–1^, n.s							
Associations	Attention and working memory, *F*(6,119) = 13.244, *p* = 4.0 × 10^–4^* Memory, *F*(6,119) = 14.129, *p* = 2.7 × 10^–4^* Language, *F*(6,119) = 8.276, *p* = 4.8 × 10^–3^* MDS-UPDRS-III, *F*(6,119) = 8.561, *p* = 4.1 × 10^–3^*							

FC network contributions, main networks, and associations with cognitive and motor functions. FC-Trait F-statistics repeated-measures ANOVA (rmANOVA) model, and the corresponding *post-hoc* between-group comparisons and associations with cognitive functions are reported.

Network presence for the FC-trait is represented by − or network not present, + or network present with medium strength, and +  + or network present with high strength. Asterisks indicate the comparisons that were statistically significant. P-values below 0.05 after FDR correction are considered statistically significant in the rmANOVA model. Between-group post-hoc tests and correlation tests with p-values below 0.01 are considered statistically significant.

The FC-trait showed a large difference between PD-MCI and both PD-CN and HC (Table 3). As depicted in the corresponding circular graph in Fig. 1A, the FC-trait connectivity is mainly driven by intra-hemispheric and inter-hemispheric connections between bilateral regions of the SMN (i.e., somatosensory, primary motor, premotor and supplementary motor areas) and the DAN and VAN. It also includes, to a lesser extent, connections between regions of the VAN, FPN and DMN networks, mainly lateralized to the left hemisphere. As depicted in the nodal strength map (Fig. 1C), somatomotor regions (i.e., bilateral precentral and postcentral gyri, as well as supplementary motor areas) are the hubs with the strongest connections in this FC-trait. Notably, the corresponding connICA weights exhibited a strong association with attention z-score and memory z-score and to a lesser extend with the language z-score, and MDS-UPDRS-III as reflected in the F-values (Fig. 1B, Table 3).

## Discussion

The present study aimed to investigate FC changes in PD-MCI that occur in the major RSN. With the present approach we have been able to identify the characteristic differences of brain connectivity between PD-MCI and cognitively normal groups (HC and PD-CN). Using the connICA framework we were able to extract independent connectivity traits from a set of individual functional connectomes that represent interactions between multiple brain systems or networks at the FC level. Our findings revealed that PD-MCI patients exhibited a distinctive FC-trait characterized by functional coupling of the SMN with the DAN, VAN and FPN. We also linked the FC-trait to cognitive and behavioral tests used for proper diagnosis and cognitive subtype classification in PD-MCI in line with the MDS criteria level II. This FC-trait was associated with high-cognitive functions such as memory and working memory as well as motor function. This extraction was done in a completely data-driven approach by maximizing subjects’ identifiability^29^, and applying ConnICA^28^.

This distinctive FC-trait indicates that altered functional coupling of the SMN in coordination with the DAN, the VAN and the FPN may be a major indicator of PD-MCI. The differential FC pattern of this FC-trait was associated with attention and working memory, memory, and language tasks, suggesting cognitive deficits in multiple domains. Importantly, these were the domains that were most negatively affected in our PD-MCI population (Table S.1), reinforcing the relevance of this trait in the early phases of cognitive decline in PD. These results are consistent with previous studies showing that PD-MCI patients displayed disrupted inter-connectivity in the DAN or the SMN and connectivity reductions between the SMN and the cognitive control network^14,23,30,31^. Although the SMN is not considered to be an association network^25^, regions belonging to this network, such as the somatosensory cortex, are crucial for higher order processes (e.g., verbal creativity)^32^. Indeed, the incidental memory test (i.e., ROCF) and the delayed recall subtest of the RAVLT, both used to assess the memory cognitive domain, have a strong sensorimotor component. The ROCF asks subjects to replicate a previously observed figure, and the RAVLT is a verbal short-term memory test. Therefore, the correlation between our FC-trait and participants’ memory scores on these tests could reflect memory recall deficits related to SMN disruptions^21,33,34^.

The attention and working memory assessments that also correlate with the observed FC-trait are representative of working memory^35–37^. FPN plays an important role in coordination with SMN in spatial working memory task performance^35,38^. Moreover, although the FPN is involved in both executive function and working memory, cognitive neuroscience studies suggest that PD mainly affects the frontoparietal areas involved in working memory whereas executive areas are preserved^39,40^. Likewise, the observed FC-trait was associated with the working memory tests corresponding to the attention and working memory domains but was not associated with tests examining executive function. The involvement of the DAN and VAN is also consistent with previous research findings. First, a negative connectivity between the SMN and DAN has been showed to adversely affect healthy subjects’ performance on tasks requiring attention and working memory^41^. Second, the DAN and VAN networks, in which cholinergic neurons play a crucial role, have been implicated in attention orienting tasks^11^. Both networks are typically compromised in PD-MCI and PD with dementia^42–44^.

Our FC-trait contains brain regions also found in MCI with AD patients. In AD-MCI the main affected brain areas include temporal, posterior parietal and hippocampus, which typically show reductions in FC or metabolic consumption^45,46^. The PD-MCI FC-trait found in the present study includes the DAN and posterior parietal areas linked to memory, language and visuospatial deficits related to the cholinergic system which, as indicated, is also affected in AD^12,47,48^. Finally, the FPN in PD-MCI was assumed to contribute to the dysexecutive cognitive profile^49^. However, some studies have also linked the FPN to memory deficits, more in line with its role in AD-MCI, where the FPN has been observed to be hypoactivated^12,50^. Notwithstanding, the main component of our FC-trait is the SMN, deterioration in which is exclusive to PD-MCI subjects since its origin comes from dopaminergic denervation resulting in executive function and working memory deficits^12,40,51^.

Finally, MDS-UPDRS-III was also associated with this distinctive FC-trait. Given that PD patients were “ON medication” during the study, this relationship might be due to the motor progression of the disease, probably in aspects that show less improvement following dopaminergic treatment^52^. Moreover, PD-MCI is associated with worse motor scores^53^, therefore this FC-trait could reflect an interaction between the motor impairments, represented by the SMN, and the cognitive deterioration, reflected in DAN, VAN and FPN. Hence, this FC-trait could be interpreted as an aberrant FC pattern between the SMN and the DAN, the VAN and the FPN in PD-MCI patients, reflecting motor and cognitive deficits related to cholinergic problems rather than dopaminergic depletion.

Although this study has revealed new insights into FC patterns in PD-MCI through the application of an innovative analytic technique (connICA), some limitations should be noted. First, all patients were “ON medication”, which has been reported to modify RSFC^54^. Nevertheless, motion in “OFF medication” PD patients undergoing fMRI has pervasive and confounding effects that it is preferable to avoid. Second, in the present work we used a static FC connectivity approach. Dynamic FC methods can be useful to obtain additional information such as dwell time or switching between networks^55^. Third, we analyzed data collected from two resting state runs with different temporal resolution (monoband with TR = 2 s vs. multiband with TR = 800 ms, and adapting the flip angle accordingly), which can raise concerns regarding measurement-to-measurement repeatability. However, these runs were acquired in the same session on each subject, all other sequence parameters were matched as closely as possible. In addition, we applied two steps to minimize differences between each subject FC matrices: (1) The signal was filtered to keep the same spectral content. (2) we maximized the subject’s identifiability^29^ which eliminates the differences between the FC matrices of the two runs. In addition we performed a repeated measures ANOVA (rmANOVA) analysis, which accounts for double measurements as demonstrated Ge et al. 2017 where rmANOVA models differentiated intra- and inter-subject variation and computed group differences, and observed largely consistent spatial patterns of test–retest reliability between the human connectome project (HCP) and Genomics Superstruct Project (GSP) samples, despite their differences in scanning site and image protocols (HCP TR = 0.7 s; GSP TR = 3 s)^56–58^. Moreover, in this study the variation between sequences was minimized as images were obtained with the same MR scanner.

In conclusion, using a data-driven methodology approach (connICA) that is optimal for examining FC interactions between RSN, we demonstrated that PD-MCI is associated with substantial RSFC changes in critical networks implicated in cognitive and motor deficits. A FC-trait was reliably extracted which reflected a distinctive functional connectivity between networks in the PD-MCI group comprising the SMN, DAN, VAN and FPN networks and was associated with cognitive performance on memory and working memory tasks, as well as with motor symptom severity. These results suggest that PD-MCI impairments can be induced by a progression of FC abnormalities present since the onset of the disease, which probably reflect dopaminergic deficits and other early events, as well as impairment in dorsal and ventral attention regions which are likely more related to cholinergic depletion^42,44,59^. The identification of these differential FC patterns contributes to improving our understanding of cognitive decline in PD and paves the way for further examinations FC differences underlying of the clinical and cognitive changes in subtypes of PD-MCI patients which could help identify risk of progression to dementia.

## Materials and methods

### Participants

Seventy-one right-handed participants including 20 PD-CN patients, 23 PD-MCI patients and 28 healthy controls (HC) were recruited at the Movement Disorders Unit at the Hospital Universitario Donostia (Donostia-San Sebastián, Spain).

PD was diagnosed according to the UK Brain Bank criteria^60^. Exclusion criteria included history of head trauma, psychiatric or neurological disorders other than PD, other major medical comorbidities, alcohol or drug dependence or abuse, and being left-handed. PD with dementia was diagnosed according to the MDS Task Force criteria^61^, and patients fulfilling these criteria were excluded as well. All participants were screened for MRI compatibility according to standard procedures. Experimental procedures were explained to participants, and written informed consent was obtained prior to study participation, according to the Declaration of Helsinki. The study was approved by the Gipuzkoa Clinical Research Ethics Committee.

### Clinical and neuropsychological evaluation

Diagnosis of PD-MCI was made according to the MDS Task Force criteria (level II category)^26^ when the following two criteria were fulfilled: (1) cognitive decline reported by either the patient or informant, or observed by the neurologist, that did not interfere significantly with the functional independence of the patient; (2) the patient scored more than 1.5 standard deviations below control values in at least two tests in the neuropsychological battery, either within a single cognitive domain or across different cognitive domains. Normative neuropsychological test values were taken from 32 healthy controls recruited among accompanying persons of PD patients. Z scores for the tests of each cognitive domain were calculated as follows: (test score—mean score of control sample)/(standard deviation of control sample). Z scores were used to diagnose PD-MCI. These were then averaged over both tests evaluating each domain to provide composite z scores that will be used latter for correlation analysis.

The Hoehn and Yahr^62^ and MDS-UPDRS part III scales^63^ were used to evaluate motor features. As gait, freezing of gait and postural stability decline are often seen along with the cognitive decline, scores for these items from MDS-UPDRS III were evaluated separately (items 3.10, 3.11 and 3.12 of the MDS-UPDRS scale)^63^, and then summed up in a new variable “MDS-UPDRS-III axial score”. The Hospital Anxiety and Depression Scale (HADS) was used to assess the presence of anxiety or depression symptoms and the 39-Item Parkinson’s Disease Questionnaire (PDQ-39) was used to evaluate health-related quality of life. Note that for HC subjects MDS-UPDRS-III, Hoehn and Yahr, and HADS scales were not acquired and therefore have a value of zero in all statistical analyses.

Neuropsychological evaluation was performed using the Mini Mental State Examination (MMSE) and Montreal Cognitive Assessment (MOCA) for global cognition and a comprehensive neuropsychological battery including two validated tests for each of the five cognitive domains (see Table 2). Attention, comprising alertness as well as working memory maintenance and manipulation, was measured by the inverse digit span memory and symbol digit modality tests^64^. Executive function, comprising cognitive flexibility, visual scanning, motor function and verbal working memory, was measured by the trail making test B and the phonemic fluency test^65,66^. Memory, comprising the ability to retrieve items both immediately (short-term) and after a delay (long-term), was measured by the immediate recall subtest of the Rey–Osterrieth Complex Figure (ROCF) test^33^ for incidental memory, as well as the delayed recall subtest of the Rey Auditory Verbal Learning Test (RAVLT)^34^. Language, comprising semantic memory, lexical access and dysnomia, was measured using the semantic fluency and Boston naming tests^67^. Visuospatial function, comprising structural knowledge and space perception, was measured by the object decision and number location tests from the Visual Object and Space Perception Battery (VOSP)^68^.

### MRI acquisition

All participants were scanned in a Siemens Trio 3 T MR-scanner with a 32-channel head coil at the Basque Center on Cognition, Brain and Language (Donostia-San Sebastián, Spain). Patients were under the first morning dose of antiparkinsonian medication during the MRI scanning session to minimize discomfort and movement^54^.

The anatomical MRI images included a T1-weighted MPRAGE (TR = 2.53 ms, TE = 3.97 ms, flip angle (FA) = 7°, field of view (FoV) = 256 × 256 mm^2^, 176 axial slices, voxel size = 1 × 1 × 1 mm^3^) and a T2-weighted Turbo Spin Echo (TR = 3.2 ms, TE = 425 ms, FA = 120°, FoV = 256 × 256 mm^2^, 176 axial slices, voxel size = 1 × 1 × 1 mm^3^). Two runs of T2*-weighted fMRI data were acquired during resting state, each with 10 min duration, with 1) a standard gradient-echo echo-planar imaging sequence (monoband) (TR = 2000 ms, TE = 29 ms, FA = 78°, matrix size = 64 × 64, voxel size = 3 × 3 × 3 mm^3^, 33 axial slices with interleaved acquisition, slice gap = 0.6 mm) and 2) a simultaneous multislice gradient-echo echo planar imaging sequence (multiband factor = 3) developed by the Center of Magnetic Resonance Research (University of Minnesota, USA) (TR = 800 ms, TE = 29 ms, FA = 60°, matrix size = 64 × 64, voxel size = 3 × 3 × 3 mm^3^, 42 axial slices with interleaved acquisition, no slice gap). Single-band reference images were also collected before the multiband resting state acquisition for head motion realignment. During both acquisitions, participants were instructed to keep their eyes open and fixate on a white cross that they saw through a mirror located on the head coil, and not to think about anything specific. Field maps were also obtained to correct field distortions.

### Data preprocessing and quality control

Automated voxel based subcortical segmentation and cortical parcellation were extracted from the T1-weighted and T2-weighted images using the FreeSurfer image analysis software (v6.0, Harvard, MA, https://surfer.nmr.mgh.harvard.edu)^69^. Anatomical parcellations were aligned to the functional space using the single-band reference image. The T1-weighted images were also warped to the Montreal Neurological Institute (MNI) template MNI152_2009, and all the spatial transformations from the subject’s space to the MNI space were collapsed in a single spatial transformation. Resting-state fMRI (RS-fMRI) data was pre-processed using AFNI^70^. First, the volumes corresponding to the initial 10 s were removed to allow the signal to achieve steady state magnetization. Subsequently, the voxel time series were despiked to reduce large amplitude deviations and slice-time corrected. Inhomogeneities caused by magnetic susceptibility were then corrected using FUGUE (FSL) using the field map images. Next, the functional images were realigned to a base volume, which was the volume with the lowest head motion for the monoband datasets and the single-band reference image for the multiband datasets. Afterwards, a single simultaneous nuisance regression step was performed, the regressors were: 6th-order Legendre polynomials; Low-pass filtering with cutoff frequency of 0.25 Hz; Six realignment parameters plus their temporal derivatives; the first five principal components of voxels in deep white matter and the first five principal components of voxels in the lateral ventricles (i.e. anatomical CompCor)^71^; the first five principal components of the brain's edge voxels^72^. The masks of white matter, lateral ventricles and edges of the brain were obtained based on the FreeSurfer tissue and brain segmentations, warped to the functional space^73^. In addition, scans potentially corrupted by artifacts were identified and censored when the Euclidean norm of the temporal derivative of realignment parameters (ENORM) was larger than 0.4 or the proportion of voxels adjusted in the despiking step exceeded 10%. Furthermore, time courses of the derivative of root mean square variance over voxels (DVARS) were also computed for each dataset^74^.

Image quality was assessed using motion plots that included grayplots before and after nuisance regression considering the previously explained motion measures^75^. Based on the censoring step, subjects with more than 20% of the volumes removed in any of the two RS-fMRI datasets were excluded. This resulted in a final sample of 23 PD-MCI, 19 PD-CN, and 21 HC participants (i.e. 63 subjects in total) whose data were included in all the analyses reported below. The ENORM and DVARS metrics after censoring did not differ statistically between the HC (ENORM mean = 0.1 ± 0.045 mm, DVARS mean = 0.025 ± 0.006), PD-CN (ENORM mean = 0.098 ± 0.041 mm; DVARS mean = 0.026 ± 0.004 mm) and PD-MCI groups (ENORM mean = 0.099 ± 0.048 mm; DVARS mean = 0.025 ± 0.005 mm); (ENORM *p* = 0.98, DVARS *p* = 0.91).

### Definition of functional connectivity matrices 

**Figure 2:**
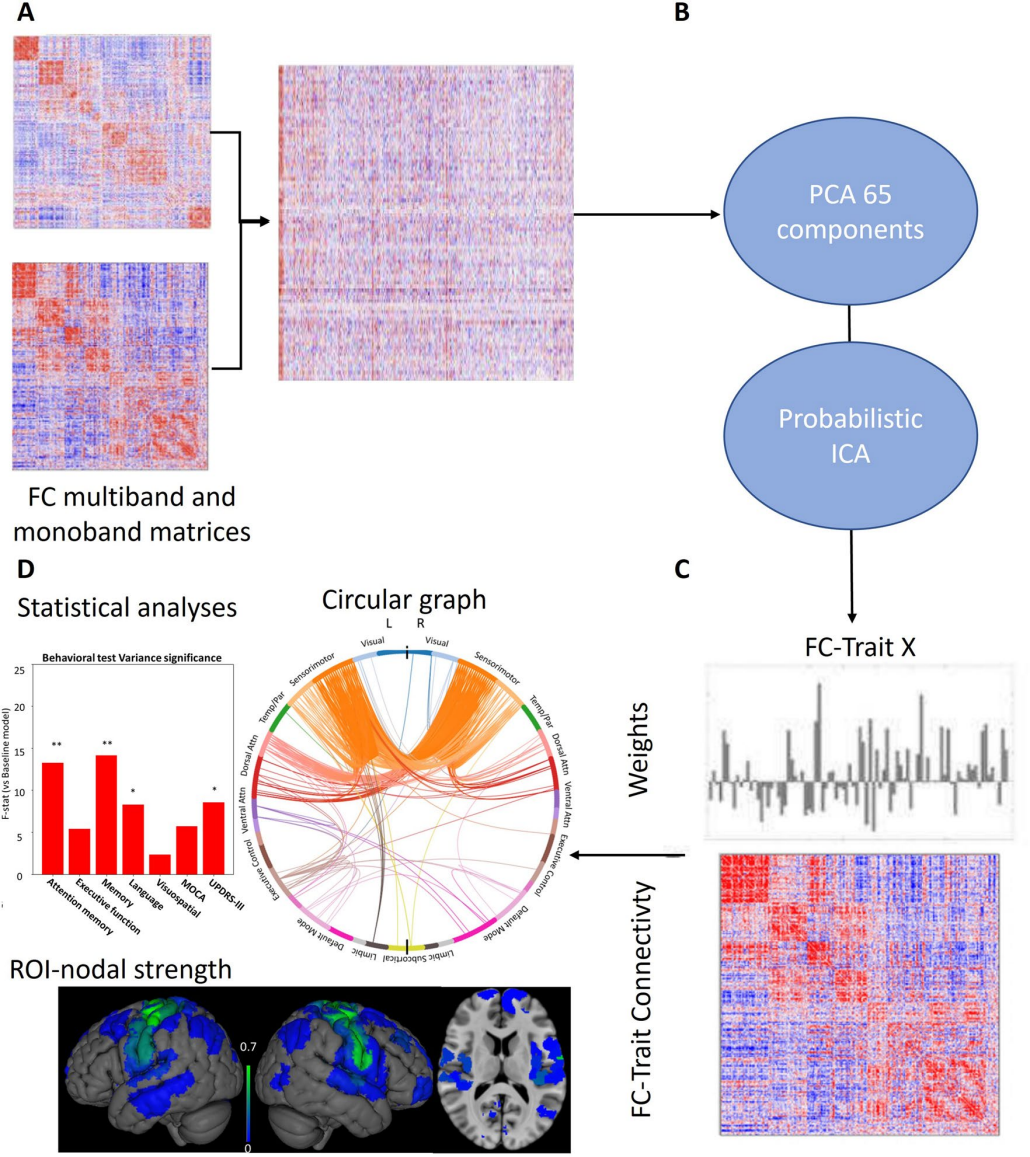
ConnICA FC-traits extraction process. (**A**) Multiband and monoband FC matrices were computed for each subject and the FC coefficients in the upper-triangular matrix were vectorized and concatenated into a group FC matrix. (**B**) The dimensionality of the group FC matrix was reduced to 65 components based on optimal subject identifiability. Then, the ConnICA decomposition was applied so that (**C**) the group FC matrix was decomposed into spatially independent functional connectivity components (a.k.a. FC-traits) and their corresponding weights for each FC matrix. (**D**) After testing for significant differences between groups, each functional trait was characterized by a circular graph, a nodal strength map and ANOVA regressions to neurophysiological assessments.

FC matrices (Fig. 2A) were obtained for each subject and each type of functional acquisition sequence (i.e. monoband and multiband) by computing the pairwise Pearson’s correlation between the average time series of ROIs defined from the 400-parcels Schaefer functional cortical atlas^76^ plus the 8 bilateral subcortical regions from the FreeSurfer brain parcellation^77^. This parcellation was chosen since we were interested in RSN such as SMN, DAN, VAN and FPN. The Schaefer functional has a one-to-one association between parcels and 17 large scale functional networks^10^. The Schaefer atlas is defined in the MNI volumetric space and was warped back to the subject’s functional space using the corresponding spatial MNI-to-functional spatial transformations, whereas the subcortical segmentations were computed in the native T1-weighted anatomical space and then also coregistered to the subject’s functional space.

### Independent FC traits

We aimed to disentangle the latent independent functional connectomes embedded in the set of subject-specific global FC matrices using a using ConnICA^28^. ConnICA is two-step process (see Fig. 2B), first a dimensionality reduction with PCA, second, an ICA to extract spatially independent pattern of functional connectivity also referred as FC-trait.

**Figure 3:**
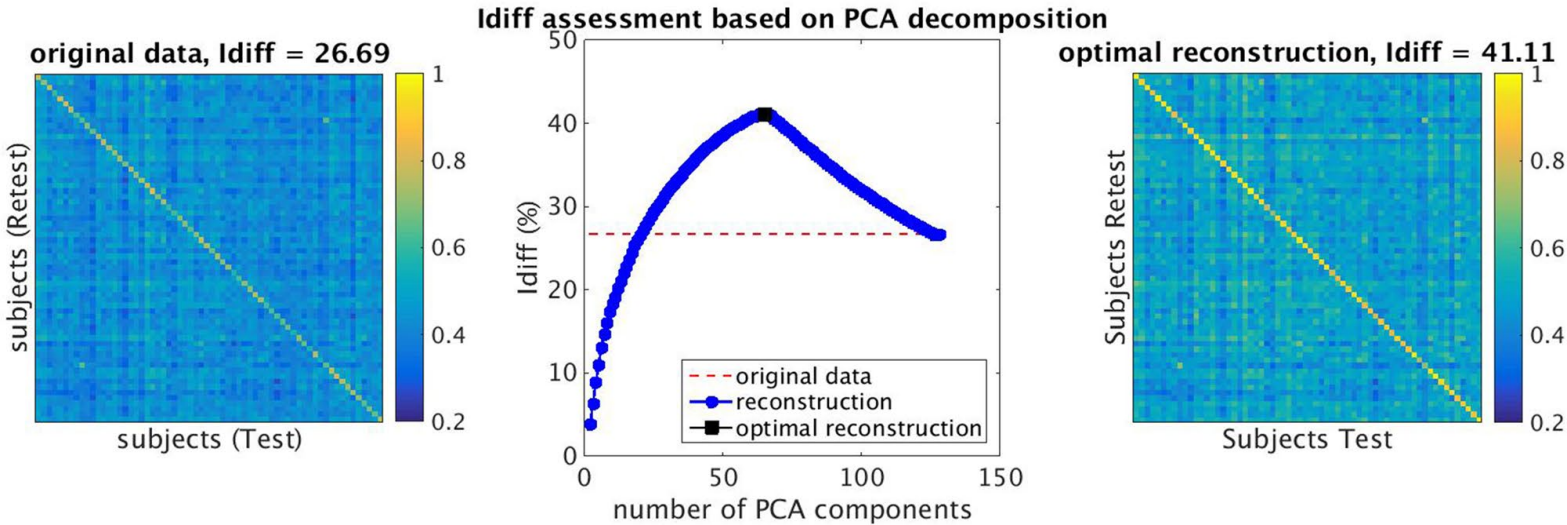
Identifiability assessment based on Principal component analysis (PCA) decomposition (**A**) Original differential identifiability (I_diff_) for our dataset was 26.69; (**B**) I_diff_ assessment computes differential identifiability for each possible increasing number of principal components (K); (**C**) After applying subject identifiability assessment, the best reconstruction was 65 components, which provided an I_diff_ of 41.11.

First, Principal Component Analysis (PCA) was performed to reduce the dimensionality of the space spanned by the FC matrices. Since the two FC matrices computed per subject were obtained from fMRI data with two different sequences (monoband and multiband), we took this further step to ensure they were compatible and to maximize the identifiability of our participants´ FC matrices by finding the PCA reconstruction that best identified each individual’s FC matrix for both the multiband and monoband sequences^29^. This approach estimates the differential identifiability (I_diff_), which quantifies the difference between the average within-subject FC similarity and the average between-subjects FC similarity in each session as a function of an increasing number of principal components. In this assessment, PCA was applied to the concatenated FC matrices for all K possible numbers of principal components; then the FC matrices were reconstructed. Finally, we selected the number of K principal components that maximized this differential identifiability; in our case, K = 65 (Fig. 3).

Next, we applied connICA^28^ (Fig. 2) to decompose the group of subject-specific functional connectomes into 65 independent functional connectomes, also referred to as FC-traits, using Probabilistic ICA^78^. Each FC-trait has two parts: (1) An FC pattern representing different functional mechanisms (2) a vector of weights indicating how present the FC-trait is in each FC matrix of the input, which can be used to see group differences and correlations with cognitive and clinical test in statistical analyses.

### Statistical analyses

One-way ANOVAs were conducted to test for between-group (HC, PD-CN and PD-MCI) differences in demographic and clinical variables. Chi-square tests were used for the categorical variables, and Mann Whgitney tests were used for non-normally distributed variables. Significant differences in age and years of education were found for the PD-MCI group and, consequently, these were entered as covariates in all subsequent statistical analyses. To rule out any interactions between FC and gray-matter (GM) volume loss we ran Freesurfer’s mri_glmfit to check group differences in GM volume and cortical thickness. This analysis revealed no significant differences in the group comparisons (PD-MCI vs. PD-CN, PD-MCI vs. HCs, PD-CN vs. HCs).

To examine group differences in the FC-traits, a rmANOVA was defined to explain the two weights for each FC-trait in each subject, with group (HC, PD-CN, PD-MCI) as the between-subjects factor, acquisition sequence (monoband, multiband) as a within-subject factor, and age, gender and TGM as covariates. Differences in the FC-traits between the groups were determined as statistically significant after controlling for multiple comparisons using the False Discovery Rate (FDR) for a q-value < 0.05. Simple effects post-hoc t-tests were then performed to examine the effects between pairs of groups.

In addition, regression one-way ANOVA analyses were performed to assess the association of the FC-traits, that reached significance in rmANOVA, with the neuropsychological and clinical evaluations. These one-way ANOVA models aimed to assess the weights of the FC-traits in terms of each of the following explanatory variables (tested individually): z-scores for the 5 cognitive domains assessed, the MOCA, the MMSE, the MDS-UPDRS-III score and the MDS-UPDRS III axial score. They were compared against a baseline model with five variables of non-interest: age, type of MR sequence (i.e., monoband vs. multiband), average value of Euclidean norm of displacement parameters (avg. ENORM), total grey matter (TGM), and total intracranial volume (eTIV). F-statistics were computed for each explanatory variable separately in comparison with the five variables of non-interest, and statistical significance was set at *P* < 0.05.

### Supplementary Information

Supplementary Table S1.

## Data Availability

Data supporting the findings of this study can be requested to the corresponding author María C. Rodríguez-Oroz.
