# Supplementary material for: Interactions between functional networks in Parkinson's disease mild cognitive impairment
Source: Sci Rep. 2023 Nov 17;13:20162. doi: 10.1038/s41598-023-46991-3 (PMC10656530; doi:10.1038/s41598-023-46991-3)
Supplement: Supplementary file 1 — Supplementary Table S1. [file 41598_2023_46991_MOESM1_ESM.docx]

**Interactions between functional networks in Parkinson's disease mild cognitive impairment**

# Supplementary materials

**Table S.1** Cognitive deficits of the PD-MCI subjects.

| **Subject** | **Nº Altered domains** | **Cognitively impaired domains** | | | | |
| --- | --- | --- | --- | --- | --- | --- |
|  |  | Attention and working memory | Executive function | Memory | Language | Visuospatial |
| 01 | 4 | yes | yes | yes | yes | no |
| 02 | 4 | yes | yes | yes | no | yes |
| 03 | 5 | yes | yes | yes | yes | yes |
| 04 | 2 | no | yes | yes | no | no |
| 05 | 5 | yes | yes | yes | yes | yes |
| 06 | 4 | yes | yes | yes | no | yes |
| 07 | 5 | yes | yes | yes | yes | yes |
| 08 | 5 | yes | yes | yes | yes | yes |
| 09 | 4 | yes | yes | yes | yes | no |
| 10 | 3 | yes | no | yes | yes | no |
| 11 | 4 | yes | yes | yes | yes | no |
| 12 | 3 | yes | yes | no | no | yes |
| 13 | 2 | yes | yes | no | no | no |
| 14 | 2 | no | yes | no | yes | no |
| 15 | 3 | yes | yes | no | no | yes |
| 16 | 2 | yes | yes | no | no | no |
| 17 | 5 | yes | yes | yes | yes | yes |
| 18 | 5 | yes | yes | yes | yes | yes |
| 19 | 3 | yes | yes | yes | no | no |
| 20 | 5 | yes | yes | yes | yes | yes |
| 21 | 3 | yes | yes | no | yes | no |
| 22 | 5 | yes | yes | yes | yes | yes |
| 23 | 4 | yes | yes | yes | no | yes |

Two neuropsychological tests were administered per domain. Affected domains were determined by at least one test having a z score ≤ 1,5 relative to healthy control subjects.
